# The expression and localization of RNase and RNase inhibitor in blood cells and vascular endothelial cells in homeostasis of the vascular system

**DOI:** 10.1371/journal.pone.0174237

**Published:** 2017-03-22

**Authors:** Ayaka Ohashi, Aya Murata, Yuichiro Cho, Shizuko Ichinose, Yuriko Sakamaki, Miwako Nishio, Osamu Hoshi, Silvia Fischer, Klaus T. Preissner, Takatoshi Koyama

**Affiliations:** 1 Laboratory Molecular Genetics of Hematology, Field of Applied Laboratory Science, Graduate School of Health Care Sciences, Tokyo Medical and Dental University, Tokyo, Japan; 2 Anatomy and Physiological Science, Field of Applied Laboratory Science, Graduate School of Health Care Sciences, Tokyo Medical and Dental University, Tokyo, Japan; 3 Instrumental Analysis Research Center, Tokyo Medical and Dental University, Tokyo, Japan; 4 Institute for Biochemistry, Medical Faculty, Justus-Liebig-Universität, Giessen, Germany; Ludwig-Maximilians-Universitat Munchen, GERMANY

## Abstract

RNA may be released from vascular cells including endothelial cells in the event of injury and in vascular disease. Extracellular RNAs have been recognized as novel procoagulant and permeability-increasing factors. Extracellular RNA may function as inflammatory host alarm signals that serve to amplify the defense mechanism, but it may provide important links to thrombus formation. Extracellular RNA is degraded by RNase. We propose that RNase and its inhibitor RNase inhibitor (RI) act as modulators of homeostasis in the vasculature to control the functions of extracellular RNA. We aimed to investigate the expression and localization of RNase 1 and RI in cells that contact blood, such as platelets, mononuclear cells, polymorphonuclear cells, and red blood cells. RNase 1 and RI expression and localization in blood cells were compared with those in the human umbilical vein endothelial cell line, EAhy926. Additionally, we further investigated the effect of thrombin on the expression of RNase 1 and RI in platelets. We used an RNase activity assay, reverse transcription-polymerase chain reaction, western blot, immunocytochemistry, transmission electron microscopy, and immunoelectron microscopy (pre- and post-embedding methods). RNase activity in the supernatant from EAhy926 cells was 50 times than in blood cells (after 60 min). RNase 1 mRNA and protein expression in EAhy926 cells was highest among the cells examined. However, RI mRNA and protein expression was similar in most cell types examined. Furthermore, we observed that RNase 1 and von Willebrand factor were partially colocalized in EAhy926 cells and platelets. In conclusion, we propose that high RNase activity is ordinarily released from endothelial cells to support anticoagulation in the vasculature. On the other hand, platelets and leukocytes within thrombi at sites of vascular injury show very low RNase activity, which may support hemostatic thrombus formation. However, activated platelets and leukocytes may accelerate pathologic thrombus formation.

## Introduction

RNA may be released from vascular cells including endothelial cells in the event of injury and in vascular disease. Extracellular RNAs have been recognized as novel procoagulant and permeability-increasing factors [1–4]. However, not all RNA species have clotting function. Sufficiently long RNAs, those composed of over 100 nucleotides, can serve as templates for the contact phase of blood coagulation [1, 2]. Furthermore, hairpin-forming RNAs appear to be more potent at activating blood coagulation [5]. Polyphosphate has also been identified as a contact phase activation factor [6, 7]. In general, polyphosphate molecules composed of over 60 phosphate residues can act as potent procoagulant agents [8]. But contact activation is the predominant prothrombotic effect of microbial long-chain polyphosphates (several hundred monomers), while physiological intermediate-chain polyphosphates (60–100 monomers) cannot induce contact activation.

Extracellular RNA may function as inflammatory host alarm signals that serve to amplify the defense mechanism, but it may provide important links to thrombus formation. Extracellular RNA is degraded by RNase. We propose that RNase and its inhibitor RNase inhibitor (RI) act as modulators of homeostasis in the vasculature to control the functions of extracellular RNA. The RNase family of proteins consists of eight members. However, only certain RNases degrade RNA [9]. In the present study, we focused on RNase 1, which exists in plasma and readily degrades RNA. Thus far, there are no studies describing the expression and localization of RNase 1 and its inhibitor in the vascular system.

We aimed to investigate the expression and localization of RNase 1 and RI in cells that contact blood, such as platelets, mononuclear cells (MNCs), polymorphonuclear cells (PMNs), and red blood cells (RBCs). We also compared the expression and localization of RNase 1 and RI in blood cells with those in the human umbilical vein endothelial cell (HUVEC) line, EAhy926. Finally, we further investigated the effect of thrombin on the expression of RNase 1 and RI in platelets.

## Materials and methods

This study was approved by the ethics committee of Tokyo Medical and Dental University (1487). Informed written consent has been obtained from the participants.

### Reagents

Polycytidylic acid potassium salt (Poly (C)) was from Sigma-Aldrich (Munich, Germany). Rabbit anti-RNase 1 polyclonal antibody (HPA001140), mouse anti-RNase inhibitor (RI) monoclonal antibody (SC-166485), and mouse anti-von Willebrand factor (VWF) monoclonal antibody (MCA127T) were from Sigma-Aldrich, Santa Cruz Biotechnology (Heidelberg, Germany), and Bio-Rad Company (Kidlington, UK), respectively. Donkey anti-rabbit IgG (H+L) coupled to Alexa fluor 488 (green) (A-21206) and goat anti-mouse IgG (H+L) coupled to Alexa fluor 555 (red) (A-21424) were from Thermo Fisher Scientific (Massachusetts, USA). 1.4 nm nanogold- goat anti rabbit IgG Fab´ fragment antibody (2004) and 1.4 nm nanogold- goat anti mouse IgG Fab´ fragment antibody (2002) were from Nanoprobes (New York, USA). Goat anti-rabbit IgG coupled to 5 nm gold (EMGAR5) and goat anti-mouse IgG+IgM (H&L) coupled to 10 nm gold (EMGAF10) were from British BioCell International (Cardiff, UK). Purified human RNase 1 (13468-H08H) and RI protein (M0307S) were from Sino Biologicals (Peking, PR China) and New England Biolabs Japan (Tokyo, Japan), respectively.

Thrombin (Japan Blood Products Organization, Tokyo, Japan) was dissolved in normal saline and added to phosphate-buffered saline (PBS) to a final concentration of 2 nM.

All reagents were of reagent grade and purchased from Wako Pure Chemicals (Osaka, Japan) unless otherwise indicated.

### Cell culture

As a model of vascular endothelial cells, we used a permanent HUVEC line, EAhy926. EAhy926 cells were kindly provided by Dr. Edgell (North Carolina University, USA) and cultured as previously described [10]. EAhy926 is a hybridoma cell line, i.e. as a fusion between HUVEC cells and the A549 adenocarcinoma cell line, but is most popularly used as a model of vascular endothelial cells in the world.

EAhy926 cells were cultured in Dulbecco’s Modified Eagle’s Medium (D-MEM) with low glucose, supplemented with 10% fetal bovine serum (FBS), 1% penicillin-streptomycin (Thermo Fisher Scientific), and maintained in a 5% CO_2_ atmosphere at 37°C.

Blood cells were collected from healthy individuals using a syringe with 109 mM sodium citrate (to isolate platelets) or with heparin sodium 10 U/mL blood (to isolate other blood cells), a tourniquet, and a 21-G needle. The whole blood was rested for 30 min after collection at room temperature 25°C as we reported previously [11] to avoid preactivation of platelets. To isolate platelets, we first centrifuged blood at 50 g for 15 min and got platelet-rich plasma. Next it was added 15% ACD-A solution and centrifuged at 1000 g for 15 min. After removing platelet-poor plasma, platelets was washed with 5 ml of Tris-EDTA saline and 750 μl of ACD-A solution and centrifuged at 1000 g for 8 min twice. Finally the washed platelets were prepared using Tyrode’s Hepes buffer by a method of Zucker [12]. MNCs were isolated by density gradient centrifugation using Ficoll-Paque PLUS (GE Healthcare, Uppsala, Sweden). Blood was doubly diluted with saline and added onto Ficoll-Paque of quarter amount of diluted solution carefully. After centrifuging at 300 g for 20 min, MNC layer was washed by double amount of saline and centrifuged at 300 g for 8 min. MNCs were washed again and suspended with saline. PMNs were collected using Polymorphprep (AXIS-SHIELD, Oslo, Norway). Blood was added on the equal volume of Polymorphprep carefully and centrifuged at 500 g for 35 min. PMN layer was washed with the same volume of double diluted PBS and normal PBS and centrifuged at 400 g for 10 min. PMNs were washed again under the same conditions and suspended with PBS. RBCs that sedimented to the bottom of tubes were also collected.

Prior to all experiments, cells were washed with PBS.

### Reverse transcription-polymerase chain reaction (RT-PCR)

Total cellular RNA was isolated using a High Pure RNA Isolation Kit (Roche Diagnostics, Mannheim, Germany) and the quantity of RNA of each cell were equalized 1000 ng. RT-PCR was performed using a PrimeScript One Step RT-PCR Kit Ver.2 (Takara, Shiga, Japan) according to the manufacturer’s instructions. cDNA derived from each cell type was amplified by 30 PCR cycles (denaturation at 94°C for 30 s, annealing at 56.4°C for 30 s, and extension at 68°C for 45 s). For visualization, isolated RNA samples were electrophoresed on 1.5% agarose gels, followed by ethidium bromide staining. Densitometric analysis of the gels were used to represent the mRNA ratios by using Scion Image PC (National Institutes of Health, USA). The results were normalized to the expression levels (E) of GAPDH and expressed as the ratio E (target)/E (GAPDH) (target = RNase 1 or RI).

### Western blotting

Platelets (3 x 10^9^ cells), RBCs (3 x 10^8^ cells), EAhy926 cells, MNCs and PMNc (3 x 10^7^ cells) were incubated for the 3 h in serum-free cell culture medium or PBS.

Cell supernatants were concentrated 15-fold using centricon tubes (Millipore, Frankfurt, Germany) with a cutoff of 10 kDa. Protein (supernatant: 10 μl (derived from equal volumes of cells)/lane, RNase 1 positive control: 10 ng (0.56 pmol)/lane, and RI positive control: 30 ng (0.61 pmol)/lane) in sample buffer (2% SDS, 20 mM Tris-HCl, pH 7.2, 20 mM dithiothreitol, 17% glycerol, and 4% bromophenol blue) was reduced at 100°C for 5 min and loaded on 10% SDS polyacrylamide gels, electrophoresed, and transferred to filter membranes. After blocking, filter membranes were incubated with polyclonal antibody against RNase1, or monoclonal antibody against RI (each diluted to 1:500 or 1:1,000) followed by incubation with horseradish peroxidase-conjugated anti-rabbit or anti-mouse IgG (each diluted to 1:1,000). Detection was performed according to the manufacturer’s instructions.

### RNase activity assay

EAhy926 cells were incubated in serum-free cell culture medium and blood cells were incubated in PBS for the indicated time periods.

RNase activity was determined as previously described [13] with minor modifications. Briefly, 100 μl of supernatant was added to 100 μl of poly (C) solution (1.0 mg/ml), 47.5 μl of RNase buffer (50 mM Tris-HCl, 130 mM NaCl, 2 mM EDTA, and 0.1 mg/ml acetylated bovine serum albumin (BSA), pH 8.0), and 2.5 μl of acetylated BSA solution (10 mg/ml) followed by 15 min incubation at 37°C. Aliquots of 100 μl were mixed with 250 μl of ice-cold 6% perchloric acid and 20 mM lanthanum chloride. Next, 100 μl of a 10 mg/ml fatty acid-free BSA solution was added and mixtures were maintained on ice for 15 min followed by centrifugation for 15 min at 16,000 g and 4°C. Substrate degradation was determined by measuring the absorbance of the supernatant at 280 nm. All activity values were normalized to the same number of cells (1 x 10^6^ cells), except platelets (1 x 10^8^ cells).

### Immunocytochemistry

For staining, cells were washed twice with PBS, fixed in 4% paraformaldehyde phosphate buffer solution overnight at 4°C, and washed three times with PBS. Samples were permeabilized with 0.2% Triton X-100 in PBS followed by 15 min incubation at room temperature. Samples were then blocked with 1% BSA for 30 min at 4°C, followed by incubation with 1:300 dilutions of the polyclonal antibody against RNase1 or monoclonal antibodies against RI or VWF overnight at 4°C. After washing with PBS, cells were incubated for 1 h at room temperature with corresponding anti-IgGs (dilution 1:500) coupled to Alexa fluor. Nuclei were stained using Hoechst stain solution (Sigma-Aldrich). A series of confocal optical sections were acquired through the cells using an ECLIPSE E600 confocal microscope and analyzed with the ACT-1 v2.63 image acquisition software (Nikon, Tokyo, Japan).

### Transmission electron microscopy

Samples were fixed in 2.5% glutaraldehyde in 0.1 M phosphate buffer (PB) for 2 h. The samples were then washed with 0.1 M PB, post-fixed in 1% osmium tetroxide (OsO_4_) buffered with 0.1 M PB for 2 h, dehydrated in a graded series of ethanol, and embedded in Epon 812. Semi-thin sections (1 μm) were prepared and stained with toluidine blue. Ultrathin sections (80 nm) were collected on copper grids, double-stained with uranyl acetate and lead citrate, and observed by transmission electron microscopy (H-7100, Hitachi, Tokyo, Japan) as previously described [14].

### Immunoelectron microscopy

For immunoelectron microscopy, a pre-embedding method was applied [15]. Platelets collected from healthy donors were fixed with 4% paraformaldehyde in 0.1 M PB for 2 h, then washed with PB and permeabilized with 0.25% saponin for 30 min. After incubation with 0.005% silver-blocking solution for 30 min, the cells were incubated overnight at 4°C with anti-RNase 1 antibody (dilution 1:50) and anti-VWF antibody (dilution 1:50). After washing with 0.005% saponin in 0.1 M PB, the cells were incubated with a mixture of 1.4 nm nanogold- goat anti rabbit IgG Fab´ fragment antibody (dilution 1:50), and a mixture of 1.4 nm nanogold- goat anti mouse IgG Fab´ fragment antibody (dilution 1:50) for 2 h at room temperature. The cells were then washed with 0.005% saponin in 0.1 M PB and fixed with 1% glutaraldehyde in 0.1 M PB for 10 min. Next, cells were washed with 50 mM glycine PB and water. After silver enhancement (HQ Silver for EM (2012), Nanoprobes), the cells were washed with water and fixed with 0.3% OsO_4_ in 0.1 M PB for 17 min, then dehydrated in 50% and 70% ethanol for 10 min each and stained with 2% uranyl acetate in 70% ethanol for 1 h at 4°C. The cells were further dehydrated with a graded series of ethanol and embedded in Epon 812. Ultrathin sections were prepared and mounted on copper grids, stained with uranyl acetate, and examined with a Hitachi H-7100 electron microscope. EAhy926 cells cultured on plastic sheets were processed in the same way except they were permeabilized with 14% glycerol and 35% sucrose in 0.1 M PB for 15 sec, and subjected to freezing and thawing in liquid nitrogen for 15 sec.

A post-embedding method was also applied for immunoelectron microscopy. The cells were fixed in 4% paraformaldehyde in 0.1 M PB for 1 h, then dehydrated and embedded in LR White resin. Ultrathin sections were prepared and mounted on nickel grids. After incubation with 1.5% normal goat serum for 30 min, the sections were incubated overnight at 4°C with anti-RNase 1 antibody (dilution 1:10) and anti-VWF antibody (dilution 1:10). After washing with PB, the sections were incubated with a mixture of a goat anti-rabbit IgG conjugated to 5-nm gold particles (dilution 1:10) and a mixture of a goat anti-mouse IgG+IgM (H&L) conjugated to 10-nm gold particles (dilution 1:10) for 3 h at room temperature. The sections were then washed with water and stained with uranyl acetate, and examined with a Hitachi H-7100 electron microscope [14].

It is established that VWF is localized in Weibel-Palade bodies (WPB), secretory vesicles in vascular endothelial cells [16], and in platelet α-granules [17]. However, the localization of RNase 1 was not previously known. Therefore, we investigated the localization of RNase 1 by immunoelectron microscopy, using the pre- and post-embedding methods.

### Statistical analysis

The nonparametric Mann-Whitney U test was used for statistical analysis. For nonparametric multiple comparisons we performed Steel test or Steel-Dwass test using GraphPad Prism 5 (GraphPad, La Jolla, CA, USA). Results were considered significantly different at p<0.05.

## Results

### RNase 1 and RI expression in supernatants and lysates from EAhy926 and blood cells

**Fig 1:**
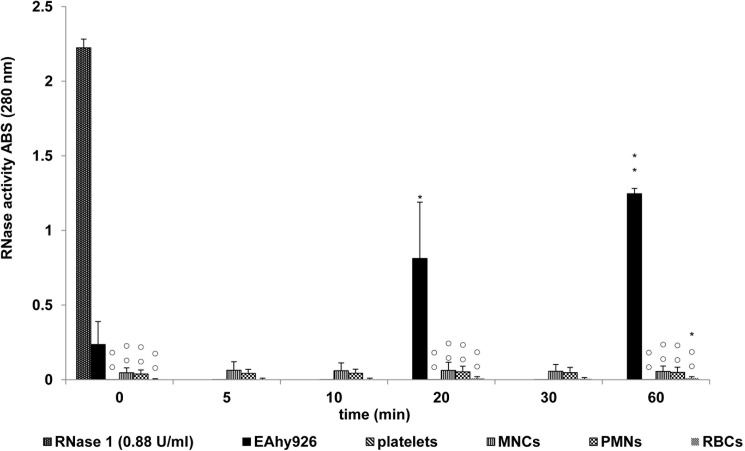
RNase activity in supernatant from EAhy926 and blood cells. RNase activity was determined in supernatant from EAhy926 cells, platelets, MNCs, PMNs, and RBCs. Each value represents the mean ± SD (n = 6). Significant differences are indicated as ○○ or ** for p<0.01. ○○ shows significant differences between EAhy926 cells and each cell type in the same time. ** shows differences between 0 min values and other min values of each cells. Whereas * indicates a significant difference with p<0.05.

To investigate the expression of RNase 1 and RI in EAhy926 and blood cells, RNase activity, and RNase 1 and RI mRNA and protein levels were determined. We examined RNase activity in the supernatants from all examined cell types (Fig 1). RNase activity in supernatants from EAhy926 cells was highest. RNase activity increased in a time-dependent manner (for 60 min from start). However, RNase activity in blood cells was low, and no time-dependent changes were observed. Cell lysates from all cells had low RNase activity (data not shown).

**Fig 2:**
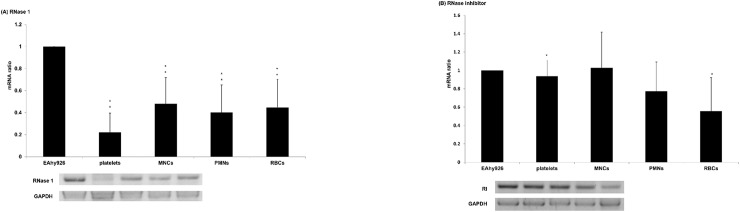
RNase 1 and RI mRNA expression in EAhy926 and blood cells. Total cellular RNA was prepared from EAhy926 and blood cells, and RT-PCR was performed as described in Materials and Methods. Densitometric analysis of the gels were used to represent the mRNA ratios by using Scion Image PC (National Institutes of Health). Results were normalized to the expression levels (E) of GAPDH and expressed as the ratio E (target)/E (GAPDH) (target = RNase 1 or RI). Each value represents the mean ± SD (n = 6) compared with EAhy926 cells. * indicates a significant differences with p<0.05, whereas ** indicates a significant difference with p<0.01.

**Fig 3:**
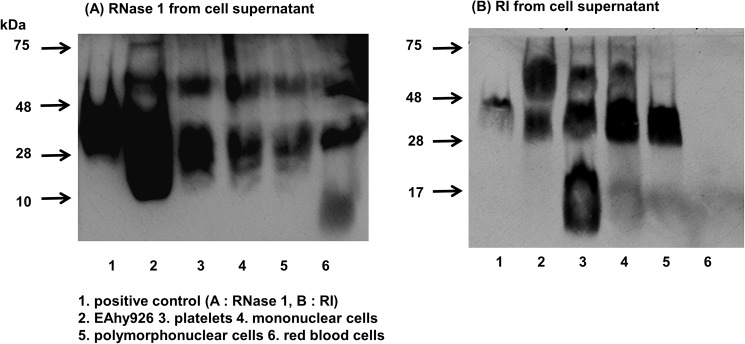
RNase 1 and RI protein expression in supernatants of EAhy926 and blood cells. (A, B) Supernatants from the different cell types were prepared and analyzed by Western blot as described in Materials and Methods with antibodies against RNase 1 (poly) and RI (mono). Lane 1. positive control (A: 10 ng (0.56 pmol) of RNase 1, B: 30 ng (0.61 pmol) of RI), 2. EAhy926 cells, 3. platelets, 4. MNCs, 5. PMNs, 6. RBCs.

We measured the mRNA and protein expression of RNase 1 and RI from EAhy926 and blood cells by RT-PCR and Western blot, respectively. RNase 1 mRNA and protein expression in EAhy926 cells was highest (Figs 2 and 3A), although RI mRNA and protein were similarly expressed in most of the cell types examined (Figs 2 and 3B). Higher bands compared with the positive control (Fig 3A and 3B) were dissociated into RNase 1 monomer plus undetermined other proteins with 8 M guanidinium chloride (data not shown).

### Localization of RNase 1 and RI in EAhy926 cells and platelets

**Fig 4:**
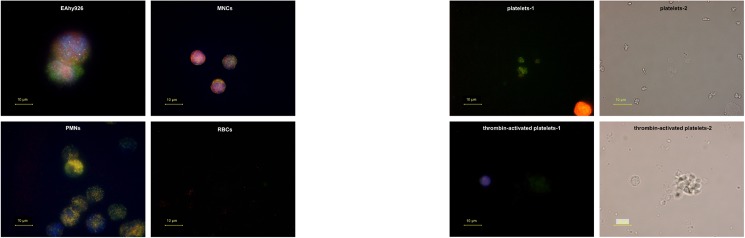
Localization of RNase 1 and RI in EAhy926 cells and platelets. After fixation and blocking, cells were stained with antibodies against RNase 1 (green), RI (red), and nuclei (blue). Each image of platelets-2 and thrombin-activated platelets-2 is optical micrograph of platelets-1 and thrombin-activated platelets-1. Scale bars = 10 μm.

The localization of RNase 1 and RI in EAhy926 cells and platelets was evaluated by immunocytochemistry. RNase 1 was highly expressed in EAhy926 cells. Furthermore, RNase 1 (green dots) and RI (red dots) in platelets activated by thrombin treatment appeared lighter than in untreated platelets (Fig 4). RNase 1 and RI were really localized within resting platelets but seemed to go out when activated.

### Localization of RNase 1 and VWF in EAhy926 cells and platelets

**Fig 5:**
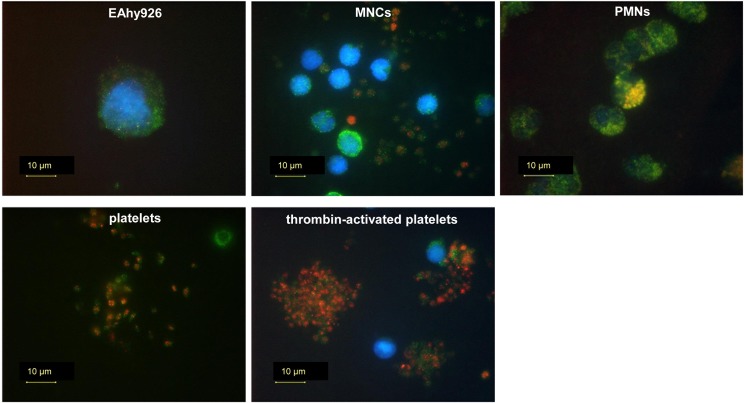
Localization of RNase 1 and VWF in EAhy926 cells and platelets. After fixation and blocking, cells were stained with antibodies against RNase 1 (green), VWF (red), and nuclei (blue). Scale bars = 10 μm.

**Fig 6:**
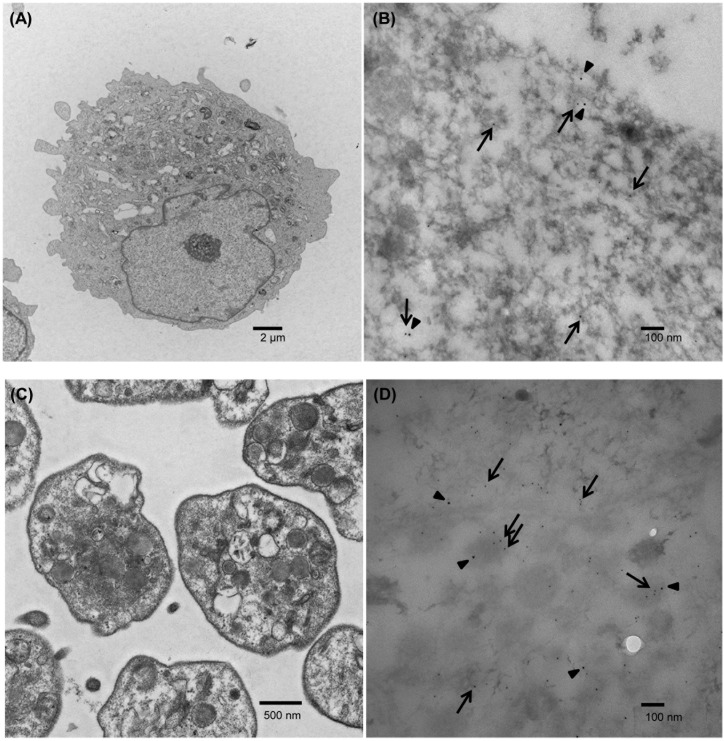
Localization of RNase 1 and VWF in EAhy926 cells and platelets by the post-embedding method. EAhy926 cells (A) or platelets (C) are TEM images. EAhy926 cells (B) or platelets (D) immunostained with anti-RNase 1 (arrows) and anti-VWF antibodies (arrowheads). Scale bars (A) 2 μm, (B) 100 nm, (C) 500 nm, (D) 100 nm.

**Fig 7:**
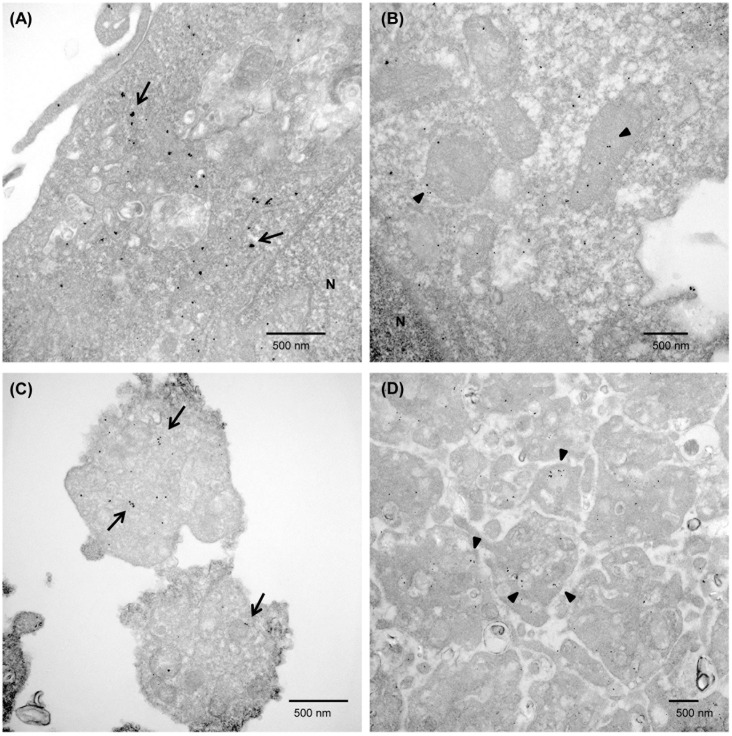
Localization of RNase 1 and VWF in EAhy926 cells and platelets by the pre-embedding method. EAhy926 cells (A, B) or platelets (C, D) immunostained with anti-RNase 1 (arrows, (A, C)) or anti-VWF antibodies (arrowheads, (B, D)). Scale bars = 500 nm.

The localization of RNase 1 and VWF in EAhy926 cells and platelets was evaluated by immunocytochemistry. RNase 1 and VWF were partially colocalized in EAhy926 cells and platelets (Fig 5). VWF is localized in WPB, secretory vesicles in vascular endothelial cells, and in platelet α-granules. Since the localization of RNase 1 was not previously known, we investigated the localization of RNase 1 by immunoelectron microscopy, using the pre- and post-embedding methods. Initially, we used the post-embedding method, which showed that RNase 1 and VWF were partially colocalized in EAhy926 cells and platelets (Fig 6). However, with this method, we could not clearly discriminate between WPBs and α-granules. For recognition of cell organelles by immunoelectron microscopy, we used the pre-embedding method. With this method, we were able to recognize organelles more clearly compared with the previous method (Fig 7).

## Discussion

We found that RNase activity in blood cells was low compared with that in EAhy926 cells. Fischer et al. reported that RNase activity was released from vascular endothelial cells [4], which was consistent with our results. And they showed a result that HUVECs expressed three times more RNase activity than EAhy926 cells. Furthermore, human pulmonary artery endothelial cells have high RNase activity. Since RI expression was higher than RNase 1 expression in blood cells [18], RNase appears to be inhibited by RI though electrostatic binding [19, 20].

We observed the expression of RNase 1 and RI mRNA in platelets and RBCs (Fig 2). Although they lack nuclei, they express RNase 1 and RI mRNA. This is attributed to the fact that mRNA is transcribed in megakaryocytes and erythroblasts, and remains in both platelets and RBCs. Platelets can also synthesize protein [21, 22]. When platelets are activated by thrombin, protein synthesis is induced [23, 24]. Therefore, we observed RNase 1 and RI protein expression from platelets stimulated with thrombin, and found that RNase 1 and RI protein were increased with higher molecular complexes (S1 Fig and S2 Fig). We examined whether RNase 1 activity was released in the supernatant of activated platelets. However, there were no significant differences in very low RNase 1 activity between activated platelets and non-activated platelets (S3 Fig). The lack of released RNase activity from activated platelets seems to be an important finding, from a hemostaseological standpoint. It appears that RNase and RI were released by degranulation, and RI almost completely inhibited the RNA-degrading activity of RNase.

The molecular weight of RNase 1 is 18–28 kDa and that of RI is 48 kDa. However, we observed bands that were larger than the sizes of RNase 1 and RI (Fig 3A and 3B). RNase and RI bind electrostatically [23]. However, since the interaction is very strong, the complex may not be dissociated by SDS-PAGE. The EAhy926 lane in Fig 3A showed a large band area below 48 kDa potentially corresponding to RNase 1 with various glycosylation degrees [25, 26], with a smaller band around 66 kDa potentially corresponding to RNase 1/RI complexes and a thin band at 75 kDa potentially corresponding to other RNase 1 complexes. The band around 66 kDa is visible in all cell types as well as on the RI blot (Fig 3B). Complex bands around 66 kDa in both, Fig 3A and Fig 3B may give a strong hint towards RNase 1/RI complexes. The intensity ratio of 18–28 kDa and higher bands of RNase 1 in supernatant of EAhy926 cells were somewhat various. Higher bands compared with the positive control (Fig 3A and 3B) were dissociated into RNase 1 monomer plus undetermined other proteins with 8 M guanidinium chloride (data not shown). We assume that RNase 1 in the supernatant binds to other proteins including RNase 1 itself [27] and RI during concentration procedure.

RNase 1 and VWF were partially colocalized in EAhy926 cells. We could not clearly discriminate WPBs, even by electron microscopy (Fig 6A). However, it is known that VWF is localized to WPBs. We therefore estimate that RNase 1 and VWF partially colocalize in WPBs. In endothelial cells, three types of exocytosis of WPBs have been proposed; single WPB exocytosis, lingering-kiss-type exocytosis, and multigranular exocytosis [16]. These mechanisms would enable the release of RNase 1. RNase 1 in platelets appeared to be partially colocalized in α-granules (Figs 6D, 7C and 7D). While RNase 1 is released from platelets through degranulation, RNase activity appears to be inhibited by the concomitant release of abundant amounts of RI. RNase 1 protein was also found in the cytoplasm of leukocytes in this study, and RNase activity appeared to be inhibited by RI.

We propose that endothelial cells usually release high RNase activity. However, at sites of vascular injury, endothelial cells are damaged, and platelets and leukocytes form thrombi which show very low RNase activity, which may support hemostatic thrombus formation. Then, activated platelets and leukocytes (i.e. at the site of inflammation) may accelerate pathologic thrombus formation [28]. Proinflammatory molecules released from platelets and leukocytes would decrease endothelial RNase1 expression and release.

## Supporting information

S1 Fig RNase 1 derived from supernatants of resting platelets and thrombin-activated platelets. Supernatants from resting and thrombin-activated platelets were prepared and analyzed by Western blot as described in Materials and Methods with antibody against RNase 1 (poly). Lane 1. positive control (10 ng (0.56 pmol) of RNase 1, B: 30 ng (0.61 pmol) of RI), 2. resting platelets, 3. thrombin-activated platelets.
(TIF)

S2 Fig RI derived from supernatants of resting platelets and thrombin-activated platelets. Supernatants from resting and thrombin-activated platelets were prepared and analyzed by Western blot as described in Materials and Methods with antibody against RI (mono). Lane 1. positive control (30 ng (0.61 pmol) of RI), 2. resting platelets, 3. thrombin-activated platelets.
(TIF)

S3 Fig RNase activity in supernatants from resting platelets and platelets activated by thrombin, ADP, or collagen. RNase activity was determined in supernatants from resting platelets and platelets activated by thrombin (2 nM), ADP (5 μM), or collagen (5 μg/ml) at indicated minutes after activation. Each value represents the mean ± SD (n = 6). ADP and collagen H were from MC Medical (Tokyo, Japan).
(TIF)
