# Supplementary figures and images for: The expression and localization of RNase and RNase inhibitor in blood cells and vascular endothelial cells in homeostasis of the vascular system
Source: PLoS One. 2017 Mar 22;12(3):e0174237. doi: 10.1371/journal.pone.0174237 (PMC5362223; doi:10.1371/journal.pone.0174237)

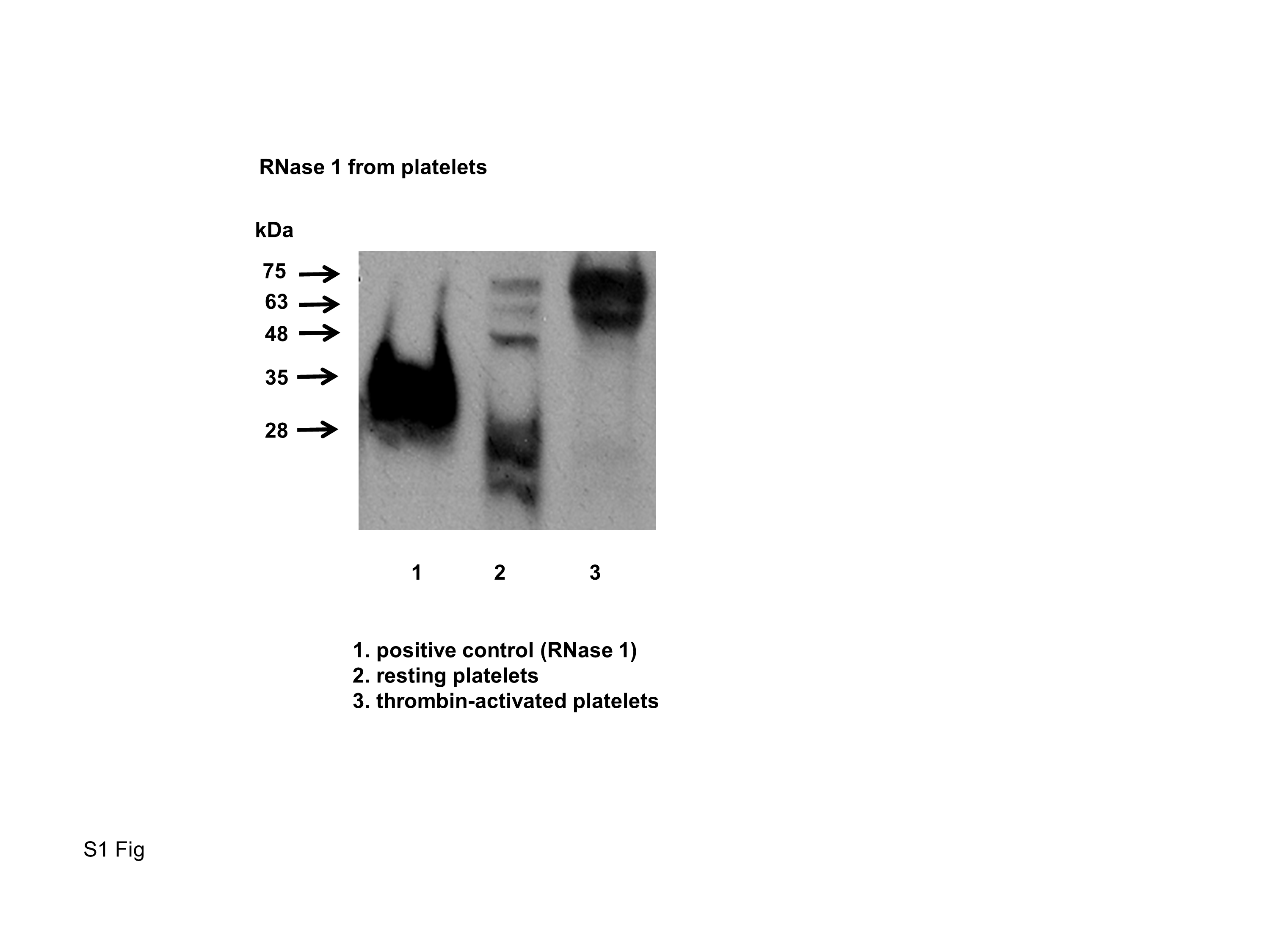

Supplement: S1 Fig — Supernatants from resting and thrombin-activated platelets were prepared and analyzed by Western blot as described in Materials and Methods with antibody against RNase 1 (poly). Lane 1. positive control (10 ng (0.56 pmol) of RNase 1, B: 30 ng (0.61 pmol) of RI), 2. resting platelets, 3. thrombin-activated platelets. (TIF) [file pone.0174237.s001.TIF]

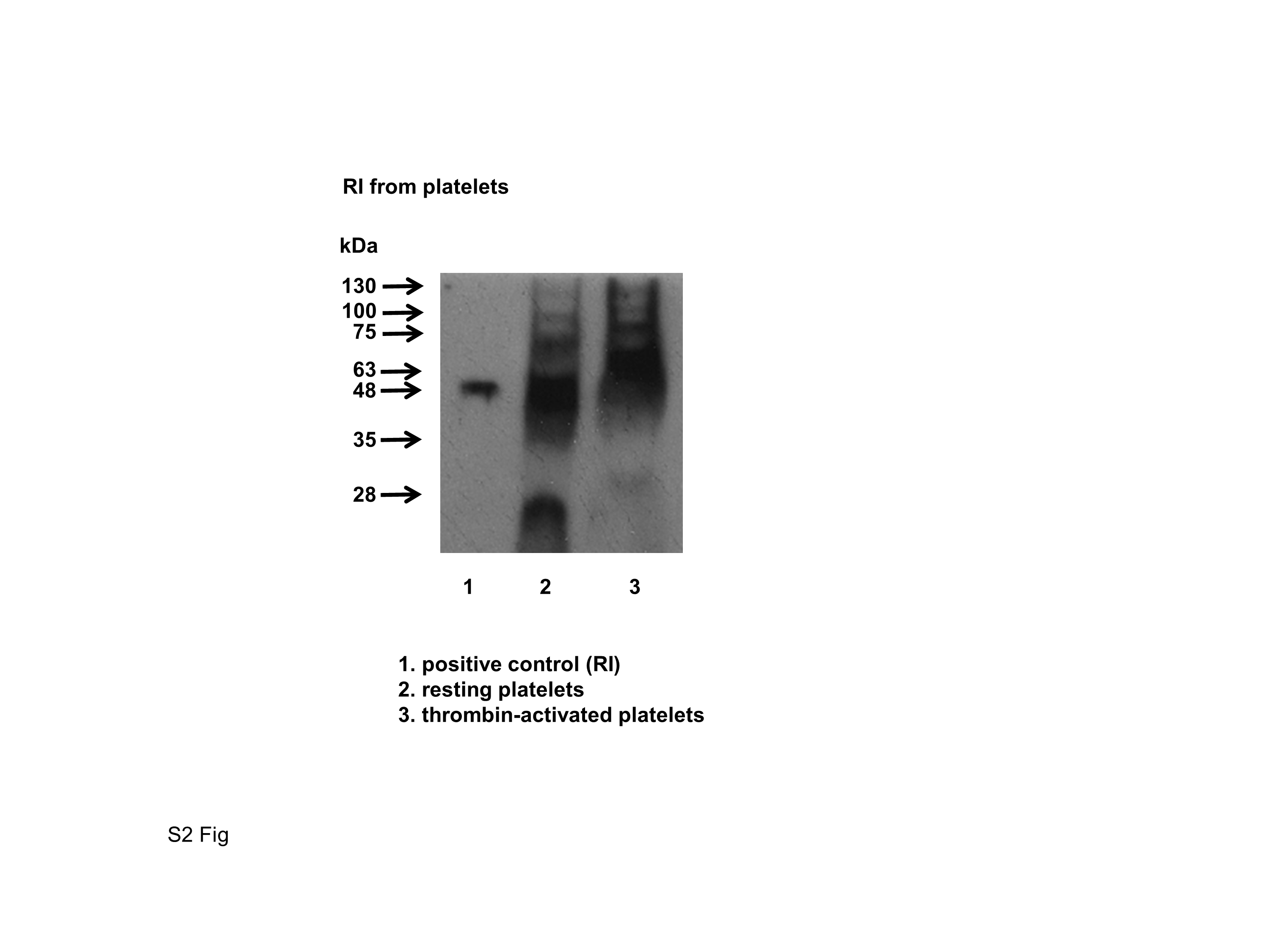

Supplement: S2 Fig — Supernatants from resting and thrombin-activated platelets were prepared and analyzed by Western blot as described in Materials and Methods with antibody against RI (mono). Lane 1. positive control (30 ng (0.61 pmol) of RI), 2. resting platelets, 3. thrombin-activated platelets. (TIF) [file pone.0174237.s002.TIF]

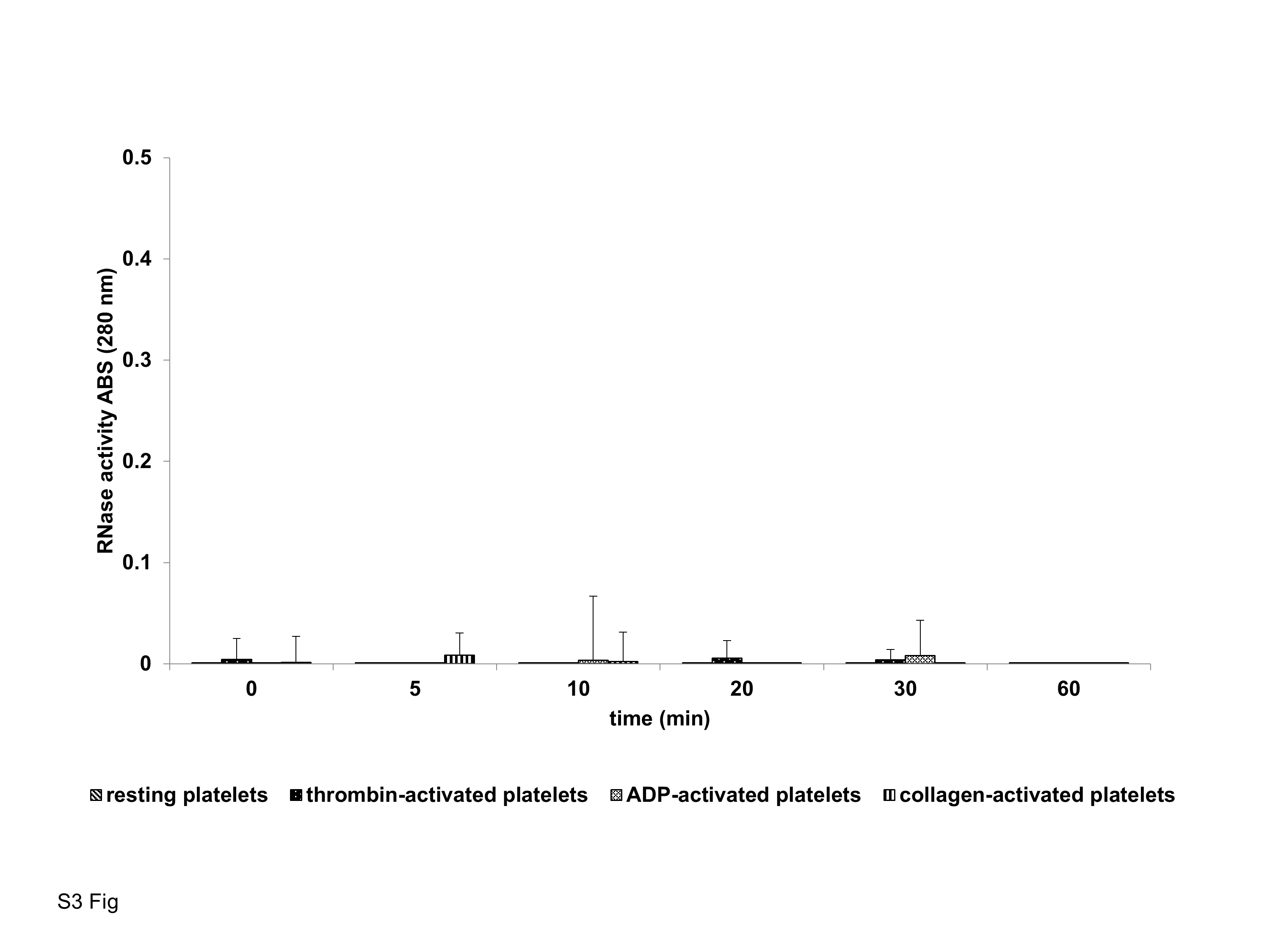

Supplement: S3 Fig — RNase activity was determined in supernatants from resting platelets and platelets activated by thrombin (2 nM), ADP (5 μM), or collagen (5 μg/ml) at indicated minutes after activation. Each value represents the mean ± SD (n = 6). ADP and collagen H were from MC Medical (Tokyo, Japan). (TIF) [file pone.0174237.s003.TIF]
